# CO_2_ Capture by Low-Cost Date Pits-Based Activated Carbon and Silica Gel

**DOI:** 10.3390/ma14143885

**Published:** 2021-07-12

**Authors:** Mohd Danish, Vijay Parthasarthy, Mohammed K. Al Mesfer

**Affiliations:** 1 Chemical Engineering Department, College of Engineering, King Khalid University, Abha 61411, Saudi Arabia; almesfer@kku.edu.sa; 2 Chemical Engineering Department, University of Petroleum and Energy Studies, Dehradun 248001, India; pvijay@ddn.upes.ac.in

**Keywords:** CO_2_ capture, capacity utilization factor, date pits, biomass, column efficiency, mass transfer zone

## Abstract

The rising levels of CO_2_ in the atmosphere are causing escalating average global temperatures. The capture of CO_2_ by adsorption has been carried out using silica gel type III and prepared activated carbon. The date pits-based activated carbon was synthesized using a tubular furnace by physical activation. The temperature of the sample was increased at 10 °C/min and the biomass was carbonized under N_2_ flow maintained continuously for 2 h at 600 °C. The activation was performed with the CO_2_ flow maintained constantly for 2 h at 600 °C. The temperature, feed flow and adsorbate volume were the parameters considered for CO_2_ adsorption. The success of CO_2_ capture was analyzed by CO_2_ uptake, efficiency based on column capacity, utilization factors and the mass transfer zone. The massively steep profiles of the breakthrough response of the AC demonstrate the satisfactory exploitation of CO_2_ uptake under the conditions of the breakthrough. The SG contributed to a maximal CO_2_ uptake of 8.61 mg/g at 298 K and C_o_ = 5% with F = 5 lpm. The enhanced CO_2_ uptake of 73.1 mg/g was achieved with a column efficiency of 0.94 for the activated carbon produced from date pits at 298 K. The AC demonstrated an improved performance with a decreased mass transfer zone of 1.20 cm with an enhanced utilization factor f = 0.97 at 298 K. This finding suggests that a date pits-based activated carbon is suitable for CO_2_ separation by adsorption from the feed mixture.

## 1. Introduction

The rising CO_2_ concentration has prompted a search for advanced expertise to minimize its influence on the environment. The utmost problem is the alarming pace at which the carbon dioxide concentration is mounting [1]. The different suggested approaches can be altered for existing plants [2,3,4] because of the fact that CO_2_ capture for post-combustion emissions is considered to be a successful process. Adsorption is the foremost technology used to separate CO_2_ from the emissions of a post-combustion process [5,6]. An adsorbent selectively adsorbs carbon dioxide and, afterwards, emits the CO_2_ that is unrelated to the process configuration [7]. The separation processes by adsorption have been primarily classified into two classes: temperature swing adsorption (TSA) and pressure swing adsorption (PSA). In the TSA process, the adsorbent is regenerated by increasing the temperature; in a PSA process, the regeneration of the used adsorbent is carried out by reducing the pressure. The vacuum pressure swing adsorption (VPSA) has been considerably examined and is used for various separation process. A study focusing on the capture CO_2_ from flue gas by adsorption at lab-scale was conducted by applying 13X zeolite as an adsorbent [8]. The findings showed that the carbon capture and sequestration (CCS) condition may be achieved by all three processes i.e., TSA, PSA and TVSA (temperature vacuum swing adsorption). An extensive review that focused on TSA and calcium looping for the sound-assisted fluidization of fine/ultrafine cohesive powders has been conducted [9]. The contribution of acoustic perturbation and its influence on the fluid dynamics of the fluidization system was found to be significant. Technologically porous adsorbents have been explored to evaluate the influence of various parameters on adsorption effectiveness [10,11,12,13,14,15,16]. The microwave irradiation technique was applied to produce activated carbon from biomass-based on a Macadamia shell [17]. A reasonable CO_2_ loading of 1.3 mmol/g was determined for the produced porous fibre [18]. The production of activated carbon by treating coal with KOH was carried out by chemical activation and an increased CO_2_ loading of 9.09 mmol/g was achieved [19]. The CO_2_ activated porous carbon has been synthesized from biomass-based on olive residues [20]. A CO_2_ capacity close to 0.58 mmol/g at 18 °C was determined for inexpensive carbon black prepared by treatment with magnetite particles [21]. In relation to temperature swing adsorption (TSA), CO_2_ adsorption has been studied using activated carbon and the findings showed that CO_2_ adsorption is affected by temperature and pressure in different ways [22]. The introduction of waste biomass into activated carbon was developed from biomass by applying the technique of hydrothermal carbonization for CO_2_ separation [23]. 

The various activation means have been applied to develop activated carbon by pyrolysis for CO_2_ separation [24]. The characteristics of activated carbon produced from biomass have been examined by one-step and two-step activation with KOH by activation at 900 °C [25]. A maximal surface area of 1597 m^2^/g was reported with enhanced adsorption characteristics for the adsorption of acetaminophen and caffeine. A study also focused on the dye adsorption using activated carbon prepared from wood waste by impregnation with KOH [26] and a surface area of 603 m^2^/g was reported. The influence of relative humidity on CO_2_ capture using microporous activated carbon developed by KOH activation and tetraethylenepentamine was examined in [27] and it was suggested that the breakthrough curve was not affected by relative humidity. CO_2_ uptake equal to 1.8 mmol/g was achieved for an activated carbon prepared by impregnation with KOH. An appropriate adsorbent was synthesized from biomass by chemical activation with potassium hydroxide [28] and a capacity equal to 5 mmol/g was determined for a KOH/hydrochar ratio of 2:1 with 1 bar pressure. The black locust-based activated carbon was produced for increased CO_2_ adsorption with potassium impregnation and a CO_2_ uptake of 7.19 mmol/g was achieved with KOH-impregnated activation [29]. An activated carbon with wheat as the biomass was produced by impregnating the biomass with KOH and a CO_2_ uptake of 5.70 mmol/g was acquired at a temperature of 0 °C at KOH/C ratio of 1:3 [30]. The carbonization of wheat bran was carried out to develop ash and equivalent materials and a 0.07 mmol/g capacity was determined for ash pellets at 25 °C [31]. 

A pomegranate peel-based adsorbent has been developed and an adsorbate capacity of 1.25 mmol/g was obtained in the case of biomass impregnated with KOH [32]. A biomass-based on different nuts was selected to synthesize the activated carbon with KOH and the capture capacity of peanut shells was up to 5.5 mmol/g [33]. A pineapple waste-derived activated carbon has also been developed and a CO_2_ uptake of 5.32 mmol/g was reported at 0 °C with 1 bar pressure [34]. N-enriched porous activated carbon has been chemically synthesized for CO_2_ capture from Procambarus Clarkii shells and the highest CO_2_ uptake 6.48 mmol/g was determined at 1 bar pressure at 0 °C [35]. 

Activation methods to develop a porous adsorbent from biomass based on an olive stone was studied and it was suggested that the existence of supplementary O_2_ groups improved the CO_2_ adsorption uptake [36]. An adsorbate capacity of 0.710 mmol/g was realized for the porous adsorbent developed from yellow tuff at 20 °C with an emphasis on process kinetics [37]. An increased CO_2_ uptake of 101.7 mg CO_2_/g at a temperature of 30 °C was determined for the biomass-based adsorbent [38]. The walnut and rapeseed mix was carbonized and, afterwards, was exposed to high temperatures to prepare the porous carbon adsorbent [39]. An activated carbon has been prepared from biomass based on a walnut shell to make the cartridge [40]. The different shells were used to prepare porous adsorbents and the developed microstructure was analyzed by SEM and FTIR [41]. Potassium hydroxide was found to be one of the more favorable agents for producing a porous adsorbent by activation with KOH from walnut shell biomass [42]. Researchers [43] synthesized the porous adsorbent from the walnut shell and investigated the separation ability of C_6_H_6_ by treating it with ZnCl_2_/H_3_PO_4_. The walnut shell biomass-based activated carbon was produced for CO_2_ adsorption and an enhanced capacity of 69.52 mg/g was achieved [44]. 

A separation capacity of 123.1 mg/g was determined for a porous adsorbent prepared from date pits by chemical activation by impregnation with KOH [45]. The date pits-based activated carbon with phosphoric acid was applied in a batch adsorber for the elimination of green dye and an adsorbate capacity of 77.8 mg/g was achieved at 25 °C [46]. A polymer-activated carbon composite was prepared by grafting polyglucoseamine on to the porous adsorbent developed from palm date-based biomass pits and a capacity of 35.0 mg/g was obtained for Cd^2+^ ions at a pH of 5.5 with a composite dose of 200 mg [47]. The porous carbon was synthesized from palm date pits by chemical activation by treatment with gaseous ammonia and it was concluded that a reduced ammonia flow and an increased bed length led to an increased breakthrough period [48]. 

The KOH and ZnCl_2_ impregnated porous carbon was prepared by date pits and a maximal uptake of 178.57 mg/g was realized for the KOH-impregnated activated carbon [49]. The H_3_PO_4_ impregnated carbon from date pits was synthesized for the removal of malachite green with a capacity of 64.7 mg/g [50]. Chemically activated carbon was developed with H_3_PO_4_ from date pits for divalent lead ions and a capacity of 102.35 mg/g was reported with a contact time of 30 min at 30 °C [51]. The porous adsorbent from date pits was synthesized by impregnating the biomass with ZnCl_2_, KOH and H_3_PO_4_ to investigate the influence on the structure of the pores [52]. It was suggested that adsorption capacity relies on the surface area, the nature of compound adsorbed and the porosity of carbon. Porous carbons have been produced by steam activation from date stones [53] and it was concluded that biomass-based date pits can be used to develop tailored O_2_ surface groups and activated carbon. A widespread review on the uses of a date pits-based biomass highlighted its numerous applications and its various synthesis techniques have been well-described [54]. 

An adsorbent has been developed from biomass-based date pits and an enhanced CO_2_ uptake was reported [55]. The date-based biomass was utilized to develop a porous adsorbent for CO_2_ adsorption and an increased capacity equal to 3.5 mmol/g was achieved [56]. A CO_2_ loading of 6.4 mmol/g was realized by applying 1 bar pressure with 0 °C for a porous adsorbent developed using chemical activation from date sheets [57]. The capture efficiency of the adsorbent obtained from the biomass of a date stone for CO_2_ separation was determined and it was suggested that the existence of pre-adsorbed H_2_O may not substantially impact the adsorption process [58]. 

A study using the volumetric method on kinetics and adsorption equilibria for silica gel at different temperatures with pressure up to 1000 KPa has been performed and the performance characteristics were compared with activated carbon [59]. It was concluded that macro-diffusion mainly influenced the adsorption of the silica gel. A continuous fixed bed adsorption for CO_2_ capture from N_2_/CO_2_ mixture was investigated using silica gel and activated carbon and an enhanced CO_2_ uptake of 39.1 mg/g was achieved for AC with a good utilization factor of 0.92 [60]. The adsorption performance for CO_2_ and CO adsorption from syngas has been analyzed using different adsorbents at 30 °C and it was shown that CO_2_ was more positively adsorbed in comparison to CO [61]. A three-bed vacuum swing adsorption (VSA) system was used to produce pure hydrogen from reformed stream methane using a silica gel adsorbent [62] and a recovery of over 90% was achieved. 

Different adsorbents, including activated carbon, zeolites, silica gel and the metal-organic framework (MOF) were used to study the efficiency of CO_2_ uptake [63] and the influence of the operating parameters on energy efficiency was analyzed. An extensive review that specifically focused on the utilization of MOF for CO_2_ capture has been presented [64]. The chemical, thermal, mechanical and hydrothermal stabilities of MOFs were reviewed along with the adsorption mechanism for CO_2_ separation. The CO_2_ capture by adsorption from a dry CH_4_/CO_2_ mixture by using zeolite, silica gel and activated carbon was investigated at a 2 bar pressure and a 0.40 mmol/g capacity was reported for the silica adsorbent [65] and the capacity increased to 1.08 mmol/g silica at a 6 bar pressure. A huge amount of biomass (date stones) can be inexpensively obtained in Saudi Arabia. There have been limited investigations into the production of porous adsorbents from the vastly available date stone-based biomass for CO_2_ capture by adsorption. The originality of the current work stems from the method used, which involves synthesizing activated carbon by applying physical activation from biomass based on date pits for CO_2_ separation and comparing the performance with silica gel that belongs to a different class of adsorbent. The proposed adsorbent will allow the possibility of utilizing the vastly available date pits-based activated carbon for CO_2_ capture to be explored and the enhanced adsorption characteristic parameters of the AC suggest that the developed activated carbon may be economically used for such an application. The performance of CO_2_ capture was analyzed in terms of various characteristic parameters using feed rate, temperature and initial adsorbate volume as operating variables. The findings suggest that a date pits-derived activated carbon may be used economically for CO_2_ capture by adsorption.

## 2. Methodology

### 2.1. Materials

Silica gel type-III (SG) with an average size of 2 mm was procured from Sigma-Aldrich (Steinheim, Germany). The date stones-based biomass was purchased from the regional market. The collected biomass was rinsed to remove dust particles. The sample was dried overnight at 110 °C. The grinder (DLC:36250, ShineBest, Shenzhen, China) was used for size reduction purposes. The milled date stone particles were then passed through a 4 mm screen (BS series). The date stone particles (DS) were stored in air-proof containers for further use.

### 2.2. Experimental Unit

**Figure 1:**
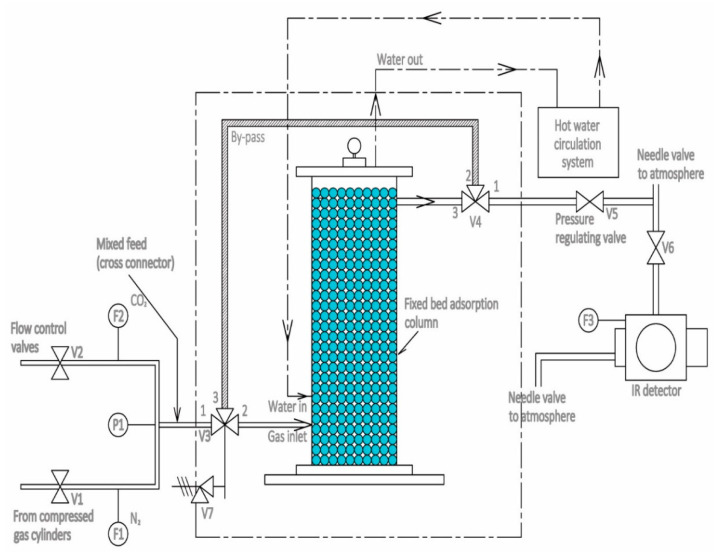
Fixed bed column [11].

The setup used for the experimental work is shown in Figure 1. The operative length of the column packed by the porous adsorbent was 24 cm. The flow rates of both the gasses were controlled by mass flow controllers. The feed composed of CO_2_/N_2_ passed into the column from the bottom side. The required flow to the IR sensor for estimating the CO_2_ concentration from the outgoing gas from the column was measured by another flow controller F3. Various thermocouples positioned inside the column were used to determine the temperature variation during the adsorption–desorption process.

### 2.3. Procedure

The mixture of CO_2_/N_2_ was allowed to enter the fixed column as per the configuration shown in Figure 1. The IR sensor measured the CO_2_ concentration at the adsorbent bed outlet at the desired regular time intervals. The uncertainty values for the measurements using F1, F2 and F3 in addition to those for the thermocouple are shown in Table 1. F1 and F2 are the mass flow controllers to measure and control the flow of N_2_ and CO_2_, respectively. The flow controller F3 measures the flow of CO_2_ to IR sensor. After the completion of the adsorption process, the desorption of the adsorbent bed was carried out by continuously flowing the N_2_ for a sufficiently long period. The complete desorption of the bed was ensured by measuring the bed outlet’s CO_2_ concentration (C) and C = 0%, which was measured up to 3 places after the decimal point before the next set of experiments were started. 

### 2.4. Physical Activation

**Figure 2:**
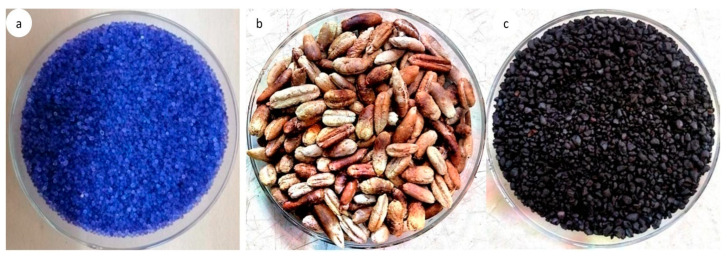
(**a**) Silica gel type III, (**b**) dried date stone and (**c**) activated carbon (AC).

The SG was for used comparative analysis as depicted in Figure 2a. A tubular furnace was applied to develop the activated carbon from the dates by physical activation. The furnace consisted of 3 zones that could be used to control dissimilar temperatures at the same time span. A sieved date stone with a known weight was placed in the inner side of the tubular furnace and afterwards, the temperature was raised at 10 °C/min with a N_2_ flow equal to 150 mL/min to reach the required temperature of 600 °C. The N_2_ was constantly maintained for an extra 2 h for carbonization. After that, the N_2_ flow was closed and the CO_2_ was allowed to flow constantly for 2 h at 600 °C to develop the desired AC. The collected date stones and the synthesized porous activated carbons are depicted in Figure 2b,c. There was a considerable reduction in the adsorbent size after the activation. The AC with an average size of 1.4 mm was produced in the experimental investigation. 

### 2.5. Adsorbent Characterization

The prepared activated carbons AC and SG were characterized using a NovaWin (Quantachrome) analyzer to determine the surface characteristics. The thermal stability of the raw date stone (DS) and the developed activated carbon (AC) were analyzed by a Thermogravimetric analyzer (Model:TG209 FI Libra, NETZSCH, Selb, Germany). The components of the activated carbon (AC) were examined by XRD (Model: PANalytical X’Pert Pro, Malvern Panalytical, Malvern, UK). The surface morphology of the selected adsorbents was analyzed by Quanta 250-FEI, SEM (Brno, Czech Republic). The Raman spectra were obtained using a Raman microscope (Model:DXR FT, Thermofisher Scientific, Berlin, Germany).

## 3. Results and Discussion

### 3.1. Morphological and Surface Area Characterisation

**Figure 3:**
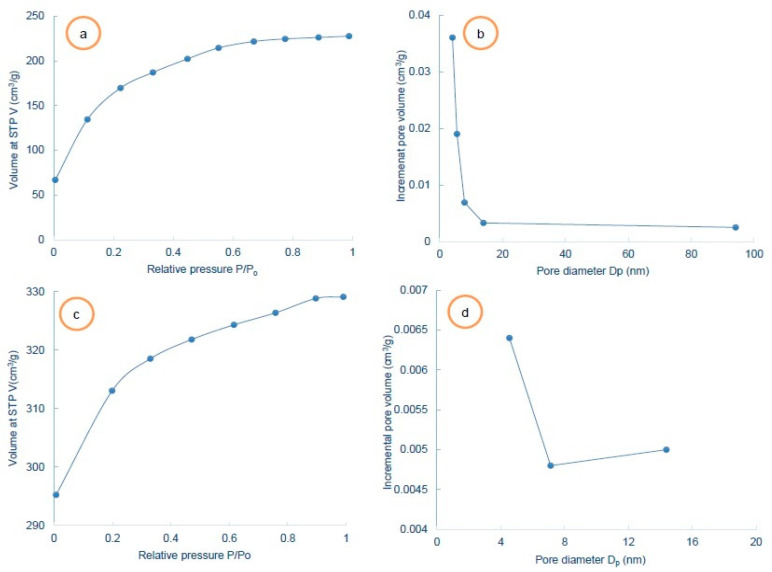
N_2_ adsorption isotherms and pore size distribution (**a**) experimental N_2_ adsorption isotherm for SG, (**b**) BJH pore size distribution for SG, (**c**) experimental adsorption isotherm for AC and (**d**) BJH pore size distribution for AC.

Synthesized activated carbons (AC) were characterized using a NovaWin (Quantachrome, Boynton Beach, FL, USA) analyzer for the surface with a 573 K outgas temperature. Prior to the measurement of the isotherms, the samples were degassed using a degasser equipped with a BET analyzer (Quantachrome Instruments, Boynton Beach, FL, USA). After reaching the required degassing temperature (523 K) using a heating metal, the degas station was selected with a vacuum degas option. The degassing of the sample was carried out for a period of 150 min. The analysis period was 88.3 min and N_2_ was supplied for analysis purposes. The surface characterisation results for AC and SG are shown in Table 2. A surface area (single point) equal to 848.27 m^2^/g was found for the synthesized activated carbon. The surface area (single point) obtained for the SG was 556.4 m^2^/g which is a smaller value compared with the surface area observed for the AC. The BET multipoint surface area obtained for the AC was also larger than that determined for the SG. Pore volumes equal to 0.45 cm^3^/g were observed for the AC which are much larger than those obtained for the SG (0.06 cm^3^/g). The pore volumes of both the adsorbents were determined by the Barrett–Joyner–Halenda method (BJH method). The pore radius of the activated carbon was significantly larger compared to that of the molecular sieve. The tabulated data show that the pore sizes for the AC and the SG were in the nm range. Due to the resolution limit of the SEM system, it was difficult to visualize the pores on the surface of the AC. However, the BET analysis confirmed that the pore size was in the nm range. Largely, the adsorbents are vastly porous materials. The sorption takes place mostly either at definite sites within the particles or on the pore walls. The experimental N_2_ isotherms and the pore size distribution (BJH method) using N_2_ gas (99.999% pure) are presented in Figure 3a–d. Figure 3a shows the N_2_ adsorption isotherm obtained up to a relative pressure (P/P_o_) of 0.99 for SG whereas Figure 3b shows the pore size distribution with the pore size in the nm scale. The experimental N_2_ adsorption isotherm for the produced carbon is depicted in Figure 3c and the pore size distribution (BJH method) is depicted in Figure 3d.

**Figure 4:**
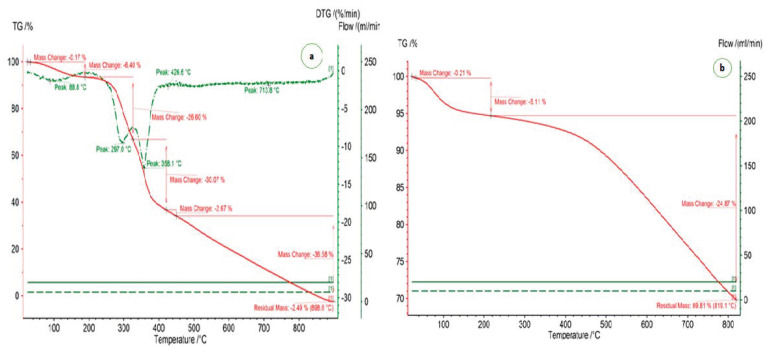
Thermogravimetric analysis of (**a**) date stone biomass and (**b**) porous AC.

Thermogravimetric instruments (Model:TG-209 FI Libra) were utilized to examine the behavior of the raw date stone and the developed activated carbon as depicted in Figure 4a,b. A sample mass (DS) of 18.4 mg was raised to 900 °C and an incremental temperature rate of 20 °C/min was fixed. The N_2_ purge gas rate was fixed at 20 mL/min. The dependence of weight loss on temperature was determined by a proximate analysis. The derivative weight change was characterized by an ostensible loss of weight. For a temperature of less than 250 °C, a 5.11% change in mass was observed. The dependence of TG (%) on temperature for the porous AC is shown in Figure 4b. A sample mass of 37.4 mg was heated up to a temperature of 800 °C and a 20 °C/min heating rate was fixed. The N_2_ flow was adjusted equal to 20 mL/min with a protective gas rate of 10 mL/min. A similar trend of minor weight loss below the activation temperature for activated carbon has been observed elsewhere [47,66].

**Figure 5:**
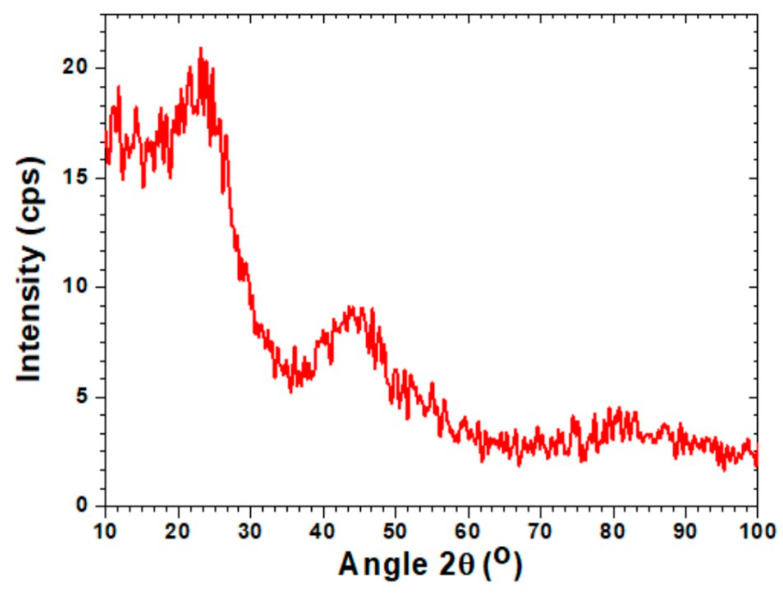
XRD analysis of synthesized activated carbon AC-SY.

The porous carbon was also examined by an XRD instrument (X’Pert Pro, Malvern Panalytical, Malvern, UK). The scan range 2θ = 0–100° was chosen to determine the X-ray patterns as depicted in Figure 5. The amorphous nature of the porous AC was identified by the existence of broader peaks. The amorphous nature of the porous carbon is a preferred characteristic for adsorbents for CO_2_ capture. Peaks at 2θ = 23.96°, 45.34° and 87.56° were observed for the carbon-based adsorbent which were analogous to the amorphous carbon peaks.

**Figure 6:**
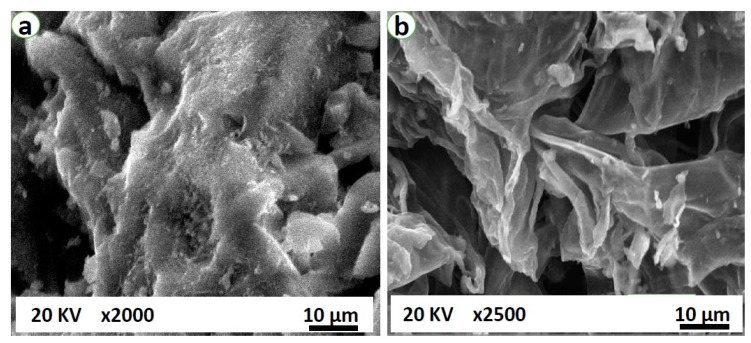
Morphological images of (**a**) silica gel type III and (**b**) the produced activated carbon.

**Figure 7:**
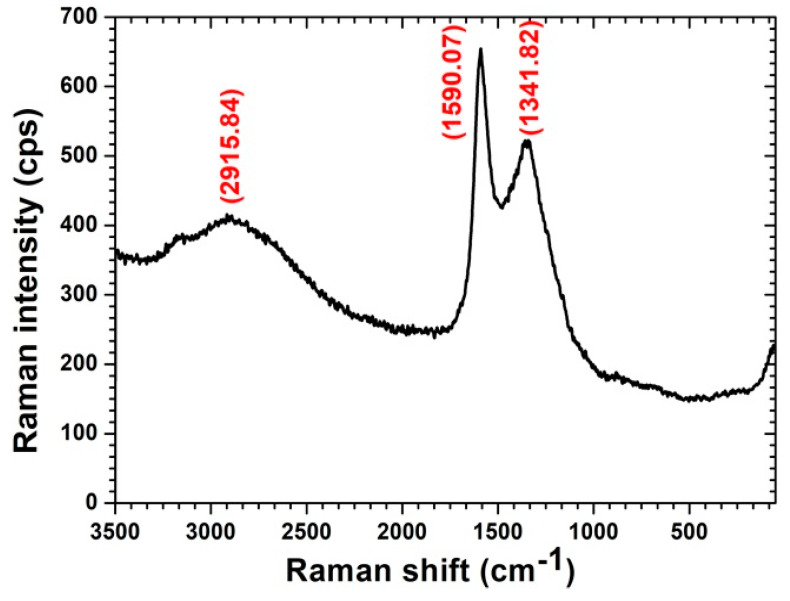
Raman spectra for the produced acrivated carbon in the spectrum range 0–3500 cm^−1^.

The surface morphology of the SG and the AC was analyzed by the SEM (Model: Quanta 250-FEI, Japan). The structural images are depicted in Figure 6a for SG and Figure 6b for AC. Many pores can be seen and the observed pores are spread all over the surface, as presented in Figure 6b with a 2500× level and Figure 6a with a 2000× level for the SG. The very regular and high density pore structures are especially effective for CO_2_ separation. The Raman spectra (Figure 7) were obtained using a Raman microscope (Model:DXR FT, Thermofisher Scientific, Berlin, Germany) equipped with excitation source of wavelength 532 nm and a laser power of 8 mW. The spectrum range of 0–3500 cm^−1^ was selected for sample scanning. The most intense bands were observed between 0 and 3500 cm^−1^. The D and G bands were observed at 1341.82 cm^−1^ and 1590.07 cm^−1^, respectively. The ratio of the intensity of the D to G bands i.e., I_D_/T_G_ specifies the extent of the defects that exist in the developed adsorbent and this ratio was found to be equal to 0.799. A smaller ID/IG ratio ensures less carbon containing defects resulting in the formation of O_2_-containing groups at the surface of the adsorbent. 

### 3.2. Breakthrough Analysis

**Figure 8:**
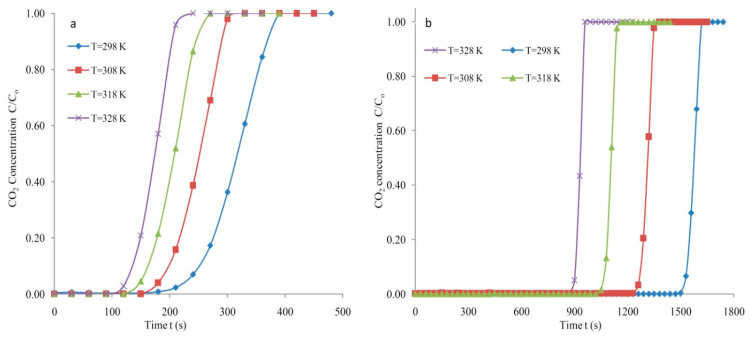
Breakthrough response at various temperatures at F = 4 lpm, C_o_ = 5% (**a**) SG and (**b**) AC.

The feed flow rates were controlled and recorded by mass flow controllers F1 and F2. The feed rate was fixed at 4 lpm and a predetermined C_o_ = 5% CO_2_ concentration level. The system pressure was adjusted equal to 1.30 bars (absolute). The adsorption curves obtained at different temperature for SG (Mass: 300 g) are depicted in Figure 8a. A breakthrough span of 224 s was determined with a saturation period of 358 s at 298 K under constant conditions of flow rate and initial CO_2_ levels. The breakpoint and exhaustion periods were reduced to 183 s and 281 s, respectively, at a higher 308 K temperature. The breakpoint period was reduced to 149 s with a saturation period of 245 s for SG with an increased temperature of 318 K. The saturation and breakthrough periods of 121 s and 197 s were determined at 328 K. It can be suggested that the breakthrough and saturation periods declined remarkably with an increased temperature. Prolonged breakthrough or exhaustion spans are crucial for a better CO_2_ uptake. Generally, an extended period (breakthrough) at a reduced temperature leads to an improved uptake of CO_2_. 

The breakthrough curves for the AC (Mass: 150 g) generated at dissimilar temperatures are presented in Figure 8b. The breakthrough time relies significantly on the temperature at which the adsorption takes place. Generally, the time it takes to reach 5% of the maximal concentration is the breakthrough time. The prolonged breakthrough and saturation periods are attributed to the reduced temperature at 298 K. The increased breakthrough periods of 1524 s were attained at the lowest temperature investigated, which was 298 K. An increased temperature of 308 K resulted in a declined breakthrough time equal to 1265 s. The breakthrough period reduced to 1059 s with an increased 318 K temperature. The maximum 328 K temperature led to an exhaustion period of 958 s. Extended exhaustion and breakthrough time spans are required for carbon-based adsorbents. The adsorption response curves produced for the AC were steeper compared to those obtained for the SG. Increased saturation and breakthrough periods were observed for the AC.

The enhanced performance of the bed for CO_2_ capture is established by the steepness of the adsorption response curve (Figure 8b). The breakthrough curves generated for the AC were relatively steep compared to the breakthrough curves obtained for the SG. The utilization of the required CO_2_ capacity is always needed to capture CO_2_ economically. The steepness of the breakthrough curve implies the thinness of the mass transfer zone. Normally, faster adsorption is attributed to a smaller MTZ. The variations in molecular weight resulted in the occurrence of adsorption. Moreover, several molecules held more firmly than others on the adsorbent surface owing to polarity. In numerous instances, the adsorbate held firmly and sufficiently to permit the inclusive capture of CO_2_ from the feed with very small or no absorption of non-adsorbables. Indeed, the degree of the steepness of the adsorption response curves presented in Figure 8b specifies a similar utilisation of capture capacity of CO_2_ at the breakthrough point. Mostly, adsorption take places above a thin zone where the concentration changes promptly. 

**Figure 9:**
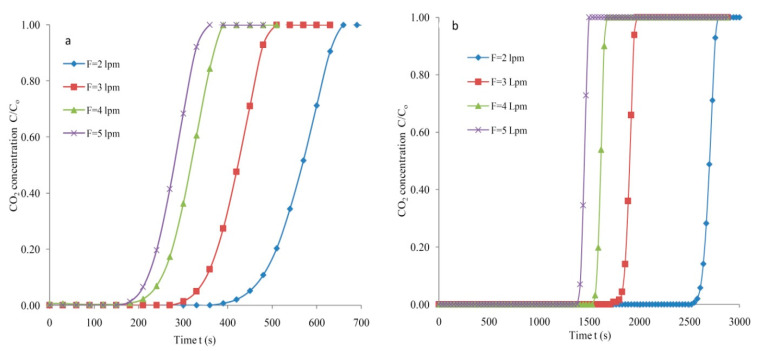
Dependence of adsorption responses on feed flow at T = 298 K, C_o_ = 5% (**a**) SG and (**b**) AC.

The dependence of the feed flow on the breakthrough period for the SG is depicted in Figure 9a. The volumetric flow rates in the range from 2 to 5 lpm varied at a constant temperature of 298 K and a 1.30 bar pressure. A flow rate of 2 lpm contributed to a breakthrough period of 447 s that is significantly lower than the period reported for the AC at the same feed mixture rate. The saturation and breakthrough times reduced to 489 s and 330 s with an increased feed flow to 3 lpm. The increased feed rate of 4 lpm led to reduced exhaustion and breakthrough spans of 381 s and 228 s with 298 K and C_o_ = 5%, respectively. The breakthrough period of 204 s was attained at an maximum feed rate of 5 lpm and T = 298 K. It can be stated that the saturation (also breakthrough) period declined with an augmented flow rate of the gaseous feed mixture.

The dependence of feed rates on the adsorption response curves generated for the AC is depicted in Figure 9b. The exhaustion and breakthrough times depend on the feed flow rates. A feed rate of 2 lpm led to extended breakthrough and saturation times of 2603 s and 2769 s. The time conforming to C/C_o_ = 0.05 was perceived to decline from 2603 s to 1833 s with varied feed rates of 2–3 lpm at 298 K. Further, the breakthrough period reduced to 1564 s with an augmented feed flow at 4 lpm. The least likely breakthrough period of 1401 s was attained for the activated carbon with a feed rate of 5 lpm. Prolonged breakthrough and exhaustion times were observed for the activated carbon compared with the SG at any predetermined feed rate. 

The adsorption response curves generated for porous carbon were very steep as mentioned earlier (Figure 9b). Furthermore, the mass transfer zones shown for the response profiles are fairly narrow, demonstrating an enhanced utilization of the CO_2_ uptake. The sharpness of the response curve is beneficial for the economical separation of CO_2_ from feed mixture. The C increases rapidly equivalent to the curve completion at the condition the adsorbent is assumed to be unproductive and this happens when the breakthrough point is attained.

**Figure 10:**
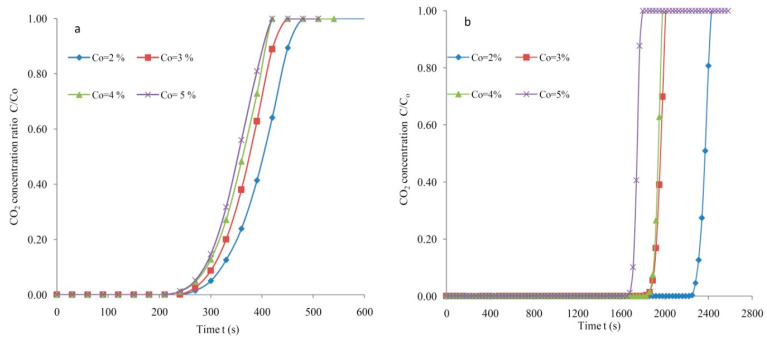
Adsorption responses at various CO_2_ levels at F = 4 lpm, T = 298K (**a**) SG and (**b**) AC.

The reliance of C_o_ on the breakthrough profiles is depicted in Figure 10a for silica gel at 4 lpm and 298K. The various CO_2_ levels in the feed were adjusted with pressures controlled at 1.30 bars. The concentration C_o_ = 2% in the feed at 298 K led to breakthrough and exhaustion periods of 300 s and 466 s with a flow rate of 4 lpm. The breakthrough and exhaustion spans were reduced to 278 s and 416 s with an increased level of the initial adsorbable gas in the feed at C_o_ = 3% level. The breakthrough period was further reduced to 270 s with an augmented C_o_ to 4% with 298 K and 4 lpm. The exhaustion period was further reduced to 392 s by increasing the CO_2_ level to 5%. The findings suggest that the breakthrough span reduces by increasing the feed rates. Prolonged breakthrough and exhaustion times are always required for an increased CO_2_ uptake. 

Adsorption response profiles at 4 lpm and 298 K reproduced at various initial adsorbate concentrations are depicted in Figure 9b for AC. The breakthrough period was reduced with a raised CO_2_ volume in the feed. The 2% CO_2_ concentration contributed to exhaustion and breakthrough periods of 2422 s and 2280 s, respectively. The breakthrough period decreased to 1884 s when the adsorbate level was increased to 3% (vol.%). A CO_2_ concentration of 4% projected the early arrival of the exhaustion period equivalent to 1976 s. A maximum adsorbate volume of 5% projected a breakthrough time of 1695 s and this demonstrates that an increased adsorbate volume causes the early arrival of the exhaustion and breakthrough periods. It was clearly observed that the exhaustion and breakthrough spans were longer for the biomass-based activated carbon compared to that determined for SG. The breakthrough span is proportional to the CO_2_ loading and varies reciprocally with the feed concentration.

### 3.3. Adsorption Capacity and Column Efficiency

The CO_2_ loading was estimated by applying the material balance. Utilising the adsorption response curves, the stoichiometric period (*t_s_*) equal to the total capacity is generally determined [67] as:

(1) $$t_{s}=\int_{0}^{\infty }\left(1-\frac{C}{C_{o}}\right)dt$$

the adsorption capacity q(mg/g) can be evaluated [68] as:

(2) $$q_{s}=\frac{F\;t_{s}C_{o}}{m_{a}}$$

where, *m_a_*: mass of the adsorbent, *F*: feed rate, *C*: adsorbate concentration at time t, *C_o_*: initial CO_2_ level in the feed.

Up to the breakthrough time *t_b_*, the time equal to the usable capacity can be calculated as:

(3) $$t_{b}=\int_{0}^{t}\left(1-\frac{C}{C_{o}}\right)dt$$

The corresponding column capacity until the breakthrough point can be calculated as:

(4) $$q_{b}=\frac{F\;t_{b}C_{o}}{m_{a}}$$

The column efficiency or the fraction of total bed capacity which is efficiently utilized (η):

(5) $$\eta =\frac{q_{b}}{q_{s}}=\frac{\int_{0}^{t}\left(1-\frac{C}{C_{o}}\right)dt}{\int_{0}^{\infty }\left(1-\frac{C}{C_{o}}\right)dt}$$

**Figure 11:**
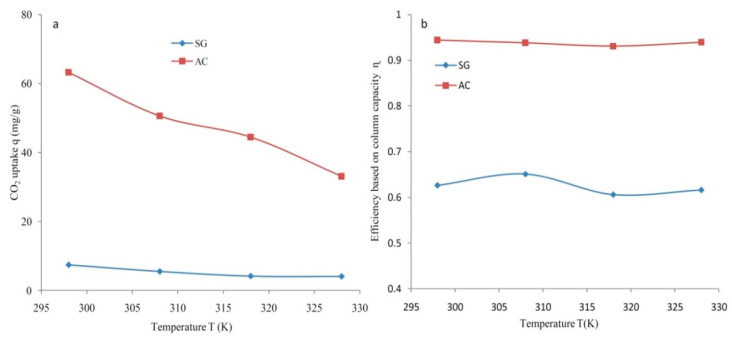
The dependence of temperature for SG and AC at C_o_ = 5%, F = 4 lpm on (**a**) CO_2_ uptake and (**b**) efficiency.

The CO_2_ uptake for the SG and the AC determined at different temperatures has been summarized in Figure 11a. The CO_2_ uptake (mg/g) varied substantially with changes in temperature for both types of adsorbents and altered negatively with changes in temperature. The maximal CO_2_ uptake (q) of 63.2 mg/g was obtained for the AC which can be compared to a lesser capacity of 7.43 mg/g for the SG at 298 K with 4 slpm. The lower CO_2_ uptake of 50.6 mg/g was acquired at an increased temperature of 308 K. The temperature of 328 K, which was the maximum studied temperature, achieved a CO_2_ uptake of 33.1 mg/g for the AC. The lowest adsorption capacity of 4.1 mg/g was exhibited by SG at the highest temperature of 328 K. All the CO_2_ capture systems predicted a reduction in the uptake with an increase in temperature. The prepared activated carbon contributed to a higher effective column efficiency (η) as evident from Figure 10b. Moreover, the column efficiency (η) based on the column capacity reported for synthesized carbon was considerably higher, with a maximum value of 0.94 at 298 K. The fraction of the total capacity that was effectively used was 0.63 for SG at 298 K. Significantly, the higher efficiency of the AC indicates its feasibility for economical CO_2_ capture. The column efficiency varied insignificantly with increased temperature for both types of adsorbents. The efficiency reported for the prepared date-based porous carbon was more than that determined for the silica gel. It can be suggested that due to an enhanced CO_2_ uptake (mg/g) and efficiency, the prepared AC performs very well and is suitable for CO_2_ separation from a CO_2_/N_2_ feed.

**Figure 12:**
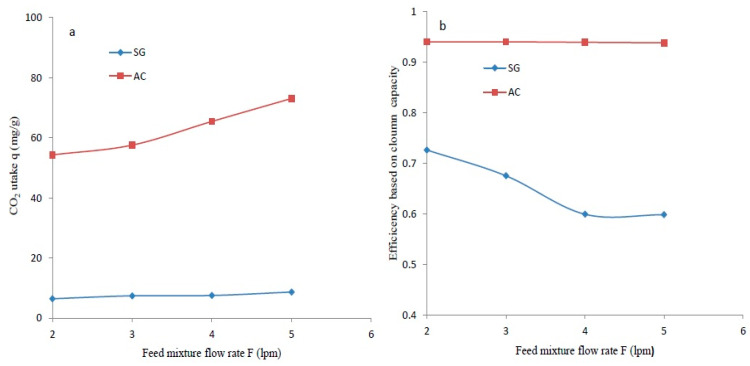
The dependence of the feed rate for SG and AC at C_o_ = 5%, F = 4 lpm on (**a**) CO_2_ uptake and (**b**) efficiency.

The adsorption performance for the SG and the AC determined at various feed flows is summarized and depicted in Figure 12. The CO_2_ uptake varied positively and substantially with feed flow for both types of adsorbents as presented in Figure 12a. The maximal CO_2_ uptake of 73.1 mg/g was determined for AC at 298 K with 5 lpm. The lowest feed flow of 2 lpm contributed to a minimal adsorption capacity and a CO_2_ uptake of 6.43 mg/g was obtained for the SG at a temperature of 298 K and C_o_ = 5%. The adsorption performance of the prepared activated carbon was reasonably higher at any fixed feed flow rate. The produced activated carbon contributed to a higher effective column efficiency. The efficiency based on column capacity determined at different flow rates is depicted in Figure 12b for SG and AC. Moreover, the efficiency obtained in the case of the synthesized carbon was considerably greater with a maximal value of 0.94 at 2 lpm or 3 lpm with 298 K and C_o_ = 5%. The highest fraction of the total capacity effectively used was 0.73 for SG with a feed flow of 2 lpm under the same conditions of temperature and initial adsorbable gas level. The average value of η = 0.94 determined for the produced carbon was considerably higher than that realized for other adsorbents (average η = 0.65). In general, it is seen that the effective column efficiency decreased marginally with an increased feed flow of 2 to 5 slpm. Significantly, the higher efficiency of AC dictates its suitability for economical CO_2_ separation from a CO_2_/N_2_ mixture. It can be predicted that an effective column efficiency varies negatively with augmented feed flow rates. It can be suggested that developed activated carbon performs very well and is suitable for CO_2_ separation from a CO_2_/N_2_ feed.

**Figure 13:**
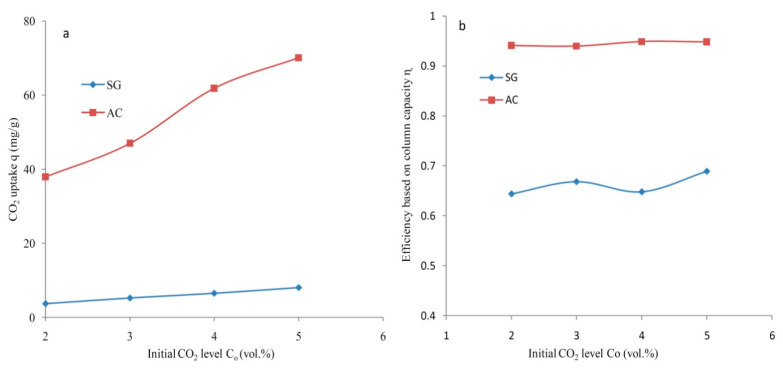
The dependence of the initial CO_2_ level for SG and AC at C_o_ = 5%, F = 4 lpm on (**a**) CO_2_ uptake and (**b**) efficiency.

The adsorption performance for the SG and the AC evaluated as a function of initial adsorbable gas concentrations has been outlined in Figure 13. The CO_2_ uptake varied largely and positively with an initial concentration level in the feed as presented in Figure 13a. The lowest and highest CO_2_ uptakes of 3.7 mg/g and 8.1 mg/g were attained for the SG at CO_2_ levels of 2% and 5%, respectively. The highest CO_2_ uptake of 70.1 mg/g was determined for the AC compared to a lower value reported for the SG at C_o_ = 5% with 298 K and a 4 lpm feed flow rate. The lowest C_o_ = 2% contributed to a minimal capacity of adsorption and a CO_2_ uptake of 37.9 mg/g was evaluated for the AC. A summary of adsorption capacity reported in literature for date stones-derived activated carbon and silica gel under different operating conditions has been presented in Table 3.

### 3.4. Mass Transfer Zone 

**Figure 14:**
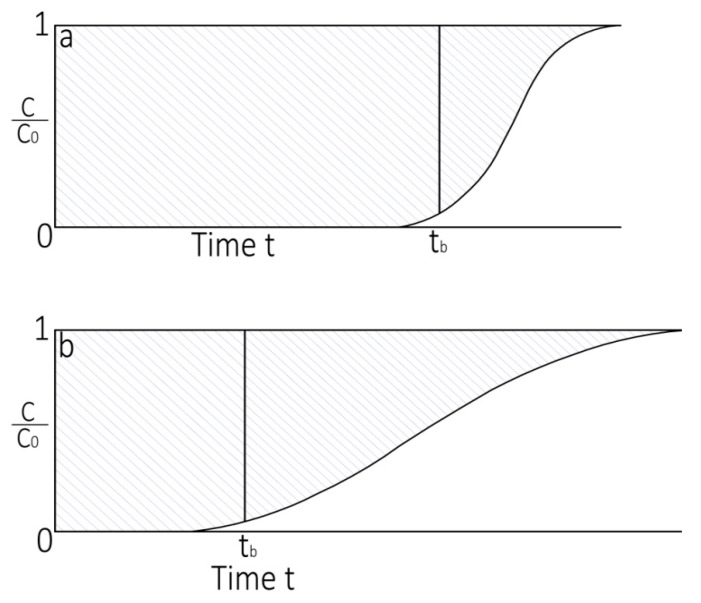
(**a**) Breakthrough profile and narrow MTZ and (**b**) breakthrough profile and wide MTZ.

The adsorbable gas amount in the solid phase and the fluid phase differ as a function of time and the location inside the column. At the earliest, the basic CO_2_ movement occurs in the vicinity of the bed input and the feed comes in to contact with a fresh porous sorbent. The CO_2_ concentration in the feed relinquishes drastically with a position effectively to zero before the end point of column is reached provided that porous adsorbent has no CO_2_ at the start of adsorption. The section of the column wherever CO_2_ is captured or the portion of the bed in which adsorbate amount varies mostly is widely known as the zone of mass transfer (*L_MTZ_*). The concentration limits are normally assumed as C/C_o_ = 5 to 95% (vol.%). A lean MTZ leads to the efficient utilization of the adsorbent resulting in a reduced regeneration cost. The MTZ usually moves from the input to the output position demonstrating that an adsorbent adjacent to the inlet position reaches the state of saturation with adsorbate gas; subsequently, the MTZ shifts towards the exit section of the bed. Figure 14a,b shows the breakthrough curves with narrow and wide mass transfer zones. The *L_MTZ_* [69] was calculated assuming a steady pattern or CO_2_ adsorption utilizing the Equation (6):

(6) $$L_{MTZ\;}=\frac{\;2\;L\left(t_{s}-t_{b\;}\right)}{t_{s}+t_{b}}$$

where, *t_s_*: exhaustion period; *t_b_*: breakthrough periods; *L*: the total bed length.

The breakthrough profile normally remains steep if the *L_MTZ_* is short relative to the total bed height and the breakthrough point leads to to the utilization of the nearly all of the bed capacity. The extended profile in the situation of the *L_MTZ_* is approximately equal to the column length. The mass transfer zone is characterized by an insignificant width assuming no axial dispersion and no resistance of mass transfer. Under such conditions, the breakthrough curve will possibly remain a vertical line between C/C_o_ = 0 and 1.0 when all the adsorbent is saturated. The porous sorbent is wholly saturated between the bed inlet and the start of the *L_MTZ_* under the breakthrough conditions. The adsorbent under the MTZ goes from nearly saturated to almost no adsorbate and for a rough average this adsorbent material is possibly believed to be nearly 50% saturated. For the assumed response profiles as uniform, the f factor may be estimated as:

(7) $$f=1-\;\frac{\;0.5\;L_{MTZ\;}}{L}$$

The bed characteristics for CO_2_ adsorption, i.e., the *L_MTZ_* and f, have been evaluated for the SG and the AC and are depicted in Table 4. The width of the mass transfer zone and the utilization factor differed marginally with elevated temperatures. A reduced *L_MTZ_* leads to an improved utilization factor. The shortest *L_MTZ_* equivalent to 7.61 cm with a utilization factor of 0.840 was tabulated at 298 K with F = 2 lpm and C_o_ = 5% for SG. It is observed clearly that the *L_MTZ_* increases with increased flow rates resulting in a reduced utilization factor. The SG type of adsorbent exhibited a maximal f = 0.875 at 298 K and F = 5 lpm with C_o_ = 5%. The feed rate variations contribute considerably to L_MTZ_ and f as evident from the findings. A *L_MTZ_* of 1.39 cm was realized at a feed flow rate of 4 lpm with a utilization factor of 0.971 at 298 K and C_o_ = 5% for the AC. The prepared activated carbon led to the minimal f = 0.955 at 298 K, F = 5 lpm and C_o_ = 5%. The f assessed at various C_o_ did vary notably under constant operating conditions of temperature and feed flow. Generally, a smaller *L_MTZ_* signifies an increased capacity factor. The nominal *L_MTZ_* of 1.25 cm suggests a respectable usage of the bed capacity at the breakthrough conditions. Generally, under different operating conditions, a smaller L_MTZ_ signifies a high utilization factor. 

**Figure 15:**
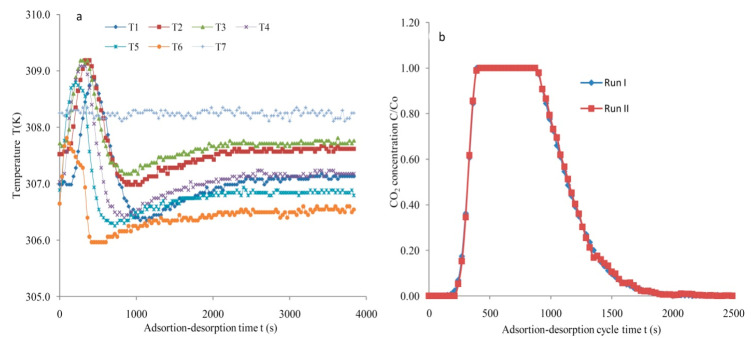
(**a**) Temperature contours for SG at F = 4 lpm and C_o_ = 5%, (**b**) repeatability assessment utilizing SG at F = 4 lpm, C_o_ = 5% and T = 308 K.

Figure 15a illustrates the profiles of the temperature recorded by sensors positioned at various locations inside the column. The data were collected for the SG type adsorbent. The temperature of the hot-water circulator was preset at 308 K using a PID controller. Temperature sensors from T1 to T6 were placed at various axial positions. The temperatures sensor had an ±0.15 K accuracy. In general, a temperature increase of about 10 (383 K) to 50 °C (323 K) may possibly occur on treating vapors with merely 1% of an adsorbable component. The temperature rise is restricted by heat loss for bed of small diameter, but a big adsorber will function approximately adiabatically. A mass transfer front is indicated by a raised temperature due to its exothermic nature and is supported by perceived profiles. Moreover, with a higher volume of adsorbate gas, the heat released during the adsorption leads to an increase in temperatures. The determination of repeatability for the silica gel is presented in Figure 15b. The data points for the two runs were used to calculate repeatability. The data were measured at 4 lpm and 308 K with an initial adsorbate concentration fixed at 5%. The robust correlation between the data sets was shown by R^2^ = 0.99. The mean error was determined as ±0.01 in terms of C/C_o_. The calculated value of σ describes the outstanding conformism. It was recommended that repeatability was amenable based on the data obtained. 

## 4. Conclusions

Dependence of temperature, feed flow and initial CO_2_ level on the breakthrough and exhaustion periods is very significant and these periods varied considerably with the operating parameters. It was clearly demonstrated that exhaustion and breakthrough spans were longer for the carbon produced from a date stone compared to that for the SG under a different set of operating conditions. The adsorption profiles produced for the AC were immensely steep, validating the outstanding utilisation of the capture capacity. The maximal CO_2_ capacity of 8.61 mg/g was reached at 5 lpm with T = 298 K and C_o_ = 5% for SG. The SG also contributed to the highest effective column efficiency of 0.689 at 298 K. A maximal CO_2_ uptake of 73.1 mg/g was determined at 5 lpm with T = 298 and C_o_ = 5 % for the AC. The activated carbon performed very well with a high column efficiency of 0.95 for bed length of 24 cm. The SG was also characterized by a minimal *L_MTZ_* = 7.6 cm at 298 K and C_o_ = 5%. The AC performed better with a reduced length of the mass transfer zone of 1.25 cm with enhanced f = 0.971 at 298 K with F = 4 lpm. It is clearly observed that synthesized carbon from economical date stone-based biomass performs better compared with the SG. It is suggested that exploiting the date stones for the synthesis of adsorbent is convincing to capture CO_2_ in a frugal way. The utilization of a date pits-derived activated carbon may contribute to environmental pollution control by adsorption-based CO_2_ capture. The current target of protecting the environment will lead to an increase in the production of these biomass-derived materials in the future.

## Figures and Tables

**Table 1:** Sensitivity of the sensors used for measurements.

Measuring Instrument	Relative Error (δu/u)
Temperature sensor	±0.0027
Flow sensor–N_2_	±0.0100
Flow sensor–CO_2_	±0.0200
IR flow sensor–CO_2_	±0.0143

**Table 2:** BET Surface characterizations of SG and AC.

Adsorbent Characteristics	SG	AC
Single point surface (m^2^/g)	556.4	848.3
Multipoint surface area (m^2^/g)	572.2	821.7
Langmuir surface area (m^2^/g)	1038.0	956.9
Pore volume (cm^3^/g)	0.06	0.45
Pore size (Å)	20.45	22.68

**Table 3:** Summary of reported CO_2_ uptakes of various adsorbents.

Adsorbent	Temperature (°C)	Pressure (bar)	Adsorption Capacity (mmol/g)	References
Date stone	20	1	3.5	[50]
Date sheet	0	1	6.4	[51]
Date sheet	25	1	4.4	[51]
Date sheet	25	40	22	[51]
Date stone	40	0.14	2.7	[52]
Silica gel	20	10	9.8	[54]
Silica gel	30	10	2.9	[56]
Silica gel	25	2	0.4	[59]
Silica gel	25	6	1.1	[59]
Date stone	25	1.3	1.7	[This study]
Silica gel	25	1.3	0.2	[This study]

**Table 4:** Characteristic parameters of CO_2_ adsorption for both adsorbents.

T (K)	F (lpm)	C_o_ (vol.%)	SG		AC	
			L_MTZ_ (cm)	f	L_MTZ_ (cm)	f
298	4	5	11.05	0.770	1.39	0.971
308	4	5	11.14	0.789	1.53	0.968
318	4	5	11.78	0.755	1.73	0.964
328	4	5	11.42	0.762	1.49	0.969
298	2	5	7.61	0.842	1.48	0.969
298	3	5	9.32	0.806	1.50	0.969
298	5	5	12.07	0.875	1.54	0.968
298	4	2	10.40	0.783	1.81	0.962
298	4	3	9.57	0.801	1.49	0.969
298	4	4	8.94	0.814	1.25	0.955

## Data Availability

Not applicable.
